# Evaluation of Dimensional Changes of 3D Printed Models After Sterilization: A Pilot Study

**DOI:** 10.2174/1874210601812010072

**Published:** 2018-01-31

**Authors:** Eman Shaheen, Abdulhadi Alhelwani, Elke Van De Casteele, Constantinus Politis, Reinhilde Jacobs

**Affiliations:** OMFS IMPATH research group, Department of Imaging & Pathology, Faculty of Medicine, KU Leuven & Oral and Maxillofacial Surgery, University Hospitals Leuven, Leuven, Belgium

**Keywords:** 3D printing, PolyJet printing, Sterilization, CAD/CAM, Splint, Tooth auto-transplantation, Surgical guide

## Abstract

**Objectives:**

To assess the effect of two of the most commonly used sterilization techniques on 3D printed clinical objects.

**Materials & Methods:**

The two sterilization methods used in our hospital and investigated in this paper are: Steam heat and Gas plasma. Three objects were printed and tested in this study: a tooth replica, an orthognathic final splint, a surgical cutting guide for the purpose of mandible reconstruction. For each of the 3 objects, 4 copies were made: one original STL object, one copy of the object pre-sterilization, one copy of post-steam heat sterilization, and one copy of post-gas plasma sterilization. Each printed object was scanned using a high resolution CBCT protocol and the compared (morphologically and volumetrically).

**Results:**

At the level of volumetric changes, no difference was found between pre and post-sterilization for both methods evaluated. As for the morphological changes, only differences were noticed with the orthognathic splint object indicating deformation of the printed splints after sterilization. Larger differences were observed with heat sterilization, making it less reliable.

**Conclusion:**

Sterilization of dental objects to be used in a clinical setting may lead to deformation of the printed model, especially for heat sterilization. Further investigations are needed to confirm these findings.

## 1 INTRODUCTION

Additive manufacturing, rapid prototyping or three-dimensional (3D) printing is a growing technology which is changing the manufacturing industry [1]. The most common techniques are Stereolithography (SLA), Selective Laser Sintering (SLS), Fused Deposition Modeling (FDM), PolyJet Technology, and Laminated Object Manufacturing (LOM). Recent developments in medical imaging along with the developments in Computer Aided Design/Computer Aided Manufacturing (CAD/CAM) allowed the fast rise of 3D printing in medicine.

Applications of 3D printing in the medical field vary from treatment planning, surgical guides, teaching models, educational tools to printing scaffolds for tissue engineering and direct printing of tissues and organs [1]. The fields of dentistry and maxillofacial surgery are also benefiting from 3D printing used in orthognathic surgery, implantology, maxillofacial reconstruction, orthodontics, prosthodontics, endodontics, *etc* [2-4].

These various clinical applications made the sterilization of the printed prototypes mandatory for their use intraoperatively. Methods used for instrument sterilization in surgical care should be reliable, practical and safe for the instruments. The main two categories are sterilization with heat and sterilization with gas [5].

The topic of sterilization of 3D printed objects for the purpose of use in the operation theatre is barely addressed [6], while sterilization of titanium in dental implants have been investigated [7].

The aim of this study was to investigate the effect of two of the most commonly used sterilization techniques on 3D printed clinical objects with a focus on PolyJet printing technology.

## 2 MATERIALS AND METHODS

### 2.1 Sterilization Methods

The two sterilization methods used in our hospital and investigated in this paper were: Steam heat and Gas plasma.

Steam heat also known as autoclave is a device used to sterilize equipment by subjecting them to high pressure saturated steam at 121°C (or higher) for around 15-20 minutes depending on the size of the load and the contents. For this study, the temperature was set to 134°C and the total duration of the cycle (including heating and cooling down) was about 60 minutes.

Sterilization with gas. Specific gases exert a lethal action on bacteria which destroys enzymes and other vital biomechanical structures. For sterilization, Ethylene Oxide (EtO) is the most commonly used but is highly flammable, needs special equipment and has a lengthy procedure to reduce tissue toxicity [5]. On the other hand, gas plasma sterilization is recommended to materials sensitive to temperature, humidity and which do not comply for EtO sterilization can be sterilized using gas plasma (vaporized hydrogen peroxide, VHP). It is a relatively new option that can provide low heat sterility cycles with none of the off-gassing concerns present with EtO. For this study, the temperature was set to 55°C and the total cycle duration was 50 minutes.

### 2.2 3D Printing Process

Three objects were printed and tested in this study:

- A tooth replica representing the process of Tooth Auto-Transplantation (TAT) [8-11].
- An orthognathic final splint representing the procedure of orthognathic surgery as described by Shaheen *et al.* [12].
- A surgical cutting guide for the purpose of mandible reconstruction [13].

Each object was selected to cover the different applications in oral and maxillofacial surgeries and was provided in Stereolithography (STL) format. Each object was printed twice in biocompatible material as it is the case in clinical practice using the PolyJet technology of Objet Connex 350 (Stratasys, Eden Prairie, MN USA) with layer thickness of 30µm. PolyJet 3D printing works similarly to inkjet printing, but instead of jetting drops of ink onto paper, PolyJet 3D printers jet layers of curable liquid photopolymer onto a build tray which are instantly cured with ultra violet light. Fine layers accumulate on the build tray to create one or several 3D models or parts [14]. Fig. (**1**) shows the original objects and Fig. (**2**) shows the 3D printed objects.

### 2.3 Validation Method

Each copy was scanned with a Cone Beam Computer Tomography (CBCT) using a high resolution protocol intended for objects and not for patients with the following acquisition settings; system: Planmeca Promax 3D Max, tube current: 12.5 mA, gray scale: 12 bits, potential: 80 kV, scan time 22.5 s, voxel size: 0.1mm, detector type: flat panel.

One copy of each object was sterilized using autoclave and the other copy of each object was sterilized using gas plasma. After sterilization, each object was scanned using the same CBCT protocol as pre-sterilization, forming 3 groups: TAT group, splint group and surgical guide group. Within each group, there are 4 copies: the original STL object, one pre-sterilization, one post-sterilization heat and one post-sterilization gas.

The DICOM (Digital Imaging and Communications in Medicine) images of each scan were exported from the CBCT and imported into Mimics Medical 19.0 (Materialise, Leuven, Belgium). The same threshold value was used to standardize the segmentation procedure for all objects to construct 3D objects which were exported from the software as STL files. These files were then imported into the 3-matic 11.0 software (Materialise, Leuven, Belgium) for further analysis.

For each group, the pre-sterilization object was registered to the original object (STL used for printing) using surface based registration. Then each post-sterilization copy was registered to the registered pre-sterilization object. Fig. (**3**) shows the example of TAT group.

We report on the volume of each object, but for more accurate morphological assessment, part comparison analysis was used. The part comparison analysis is also known as color distance map where the distance is calculated at each point on the surface of an object and the corresponding point at the surface of another object. The mean, standard deviation and Root Mean Square (R,M,S) were reported between the following:

- Pre-sterilization and the original object: to assess the deviation of the printing and scanning procedures from the original STL
- Post-sterilization heat and the pre-sterilization object: to assess the deviations resulting from the autoclave sterilization
- Post-sterilization gas and the pre-sterilization object: to assess the deviations resulting from the gas plasma sterilization.

## 3 RESULTS

### 3.1 Volumetric Assessment

Table **1** shows the results of the volume per group and the percentage change (+ indicates an increase and indicates a decrease in volume). For the accuracy test of the printing and the scanning procedures, the percentage change was between 0% to 1.8% increase in volume indicating a high accurate procedure. As for the accuracy of the heat sterilization, the percentage change in volume ranged from -0.6% to 1.5%. For the gas sterilization, the volume percent change ranged from 0.6% to 0.9% indicating that for both methods no significant change was reported.

### 3.2 Morphological Assessment

 Table **2** shows the results of the part comparison analysis. The differences reported were all below 0.1mm. The results of the splint group was not reported due to the failure of complete registration of both post-sterilized objects to the pre-sterilized one as shown in Fig. (**4**). Therefore, the registration was repeated and based on the middle part of the splint instead of a global surface registration to allow the assessment of deformation at the side parts.

In order to assess the changes occurred post-sterilization for the splint object, another method was used as shown in Fig. (**5**). A curve was drawn on the pre-sterilized splint and 7 landmarks (points) were indicated. The same landmarks were indicated on the corresponding positions once on the post-sterilization heat splint and another on the post-sterilization gas splint. The distances error were measured and presented in Table **3**.

## 4 DISCUSSION

In this study, we evaluated the effect of sterilization methods used in our hospital set-up on 3D printed surgical objects. The two most commonly used sterilization techniques are steam heat (autoclave) and gas plasma which were investigated in this paper. Whether the sterilization has an effect on 3D printed object or not is a topic not really addressed. Surgical guides especially in the field of oral and maxillofacial surgery are extensively used due to the presence of several CAD/CAM tools and low cost 3D printers. In this paper, we focused on the PolyJet technology with the Objet Connex 3D printer (the biocompatible material) since it has higher accuracy when compared to other technologies [3] and has been evaluated for biocompatibility in accordance with the industrial standard.

The results in this study showed that the percentage changes in volumes are negligible and were up to 1.5% increase in volume. As for the morphological assessment, the deformity of the objects after sterilization was also minor for the TAT and the surgical guide with a maximum mean error of 0.014mm. Although the volume changes for the splint objects after both the heat and gas sterilization was negligible, but the registration procedure failed due to the noticeable deformity in the objects after sterilization as shown in Fig. (**4**). Such deformities made the splints unusable and inaccurate for the operation. Further investigations showed that the curvature of the splints was decreased compared to the pre-sterilization situation with larger differences at the borders (points 1 and 7) with heat sterilization compared to gas sterilization (Table **3**). The heat sterilization had a maximum difference of 1.7mm while the gas sterilization had a maximum difference of 0.8 mm.

These findings indicate the necessity of further investigations on the effect of sterilization especially the heat on different 3D printing technologies and materials used as surgical guides. Furthermore, these findings are yet to be confirmed since they are not conclusive due to the small sample size, therefore, for future work we would like to enlarge the sample size and include different printing technologies with complex shapes and sizes.

## CONCLUSION

The effect of heat and gas plasma sterilization on 3D printed surgical objects with biocompatible photopolymer material was investigated. At the level of volumetric changes, no difference was found between pre and post-sterilization for both methods evaluated. As for the morphological changes, only differences were noticed with the orthognathic splint object indicating deformity of the splints after sterilization. Larger differences were observed with the heat sterilization, making it less reliable. Further investigations are needed to confirm these findings.

## Figures and Tables

**Fig. (1):**
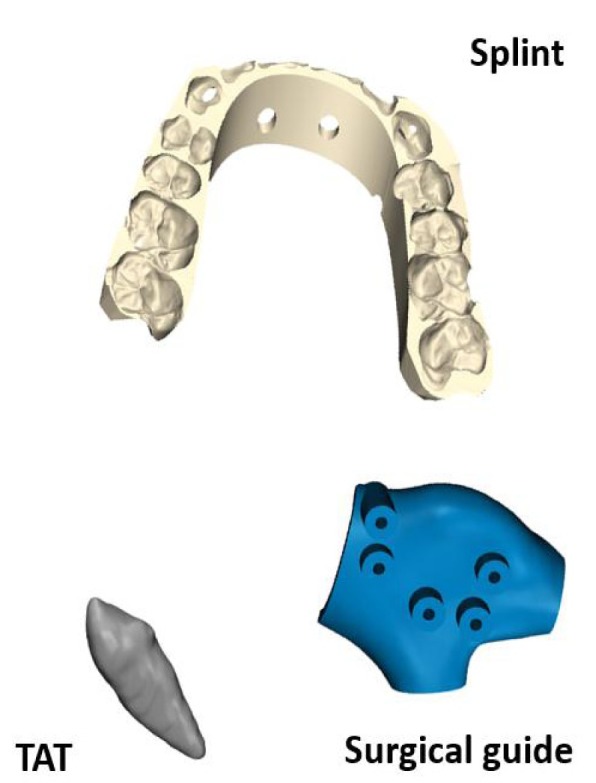
The original objects in STL format: Orthognathic splint, surgical guide and TAT.

**Fig. (2):**
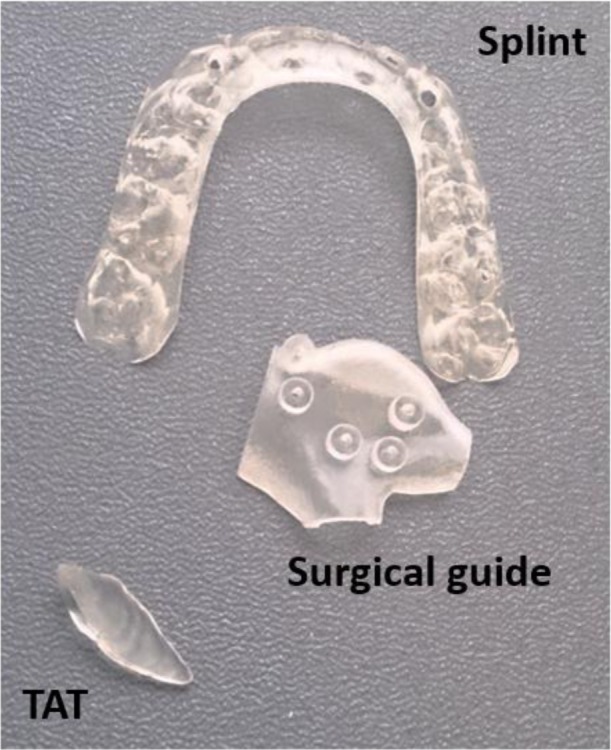
The 3D printed objects: Orthognathic splint, surgical guide and TAT.

**Fig. (3):**
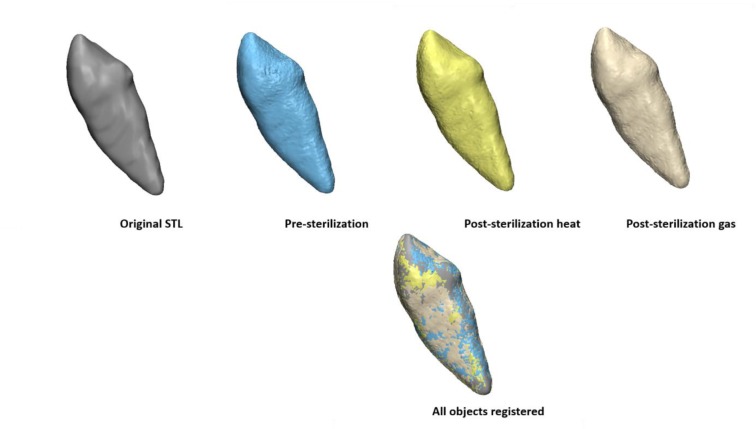
The TAT group: original STL, pre-sterilization, post-sterilization heat, post-sterilization gas and all objects registered to the original STL.

**Fig. (4):**
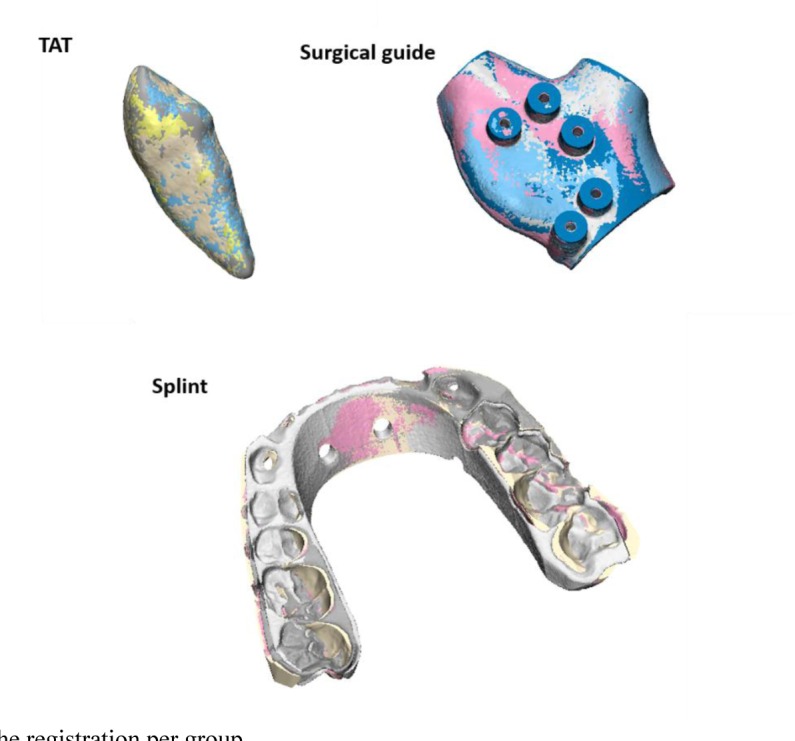
The outcome of the registration per group.

**Fig. (5):**
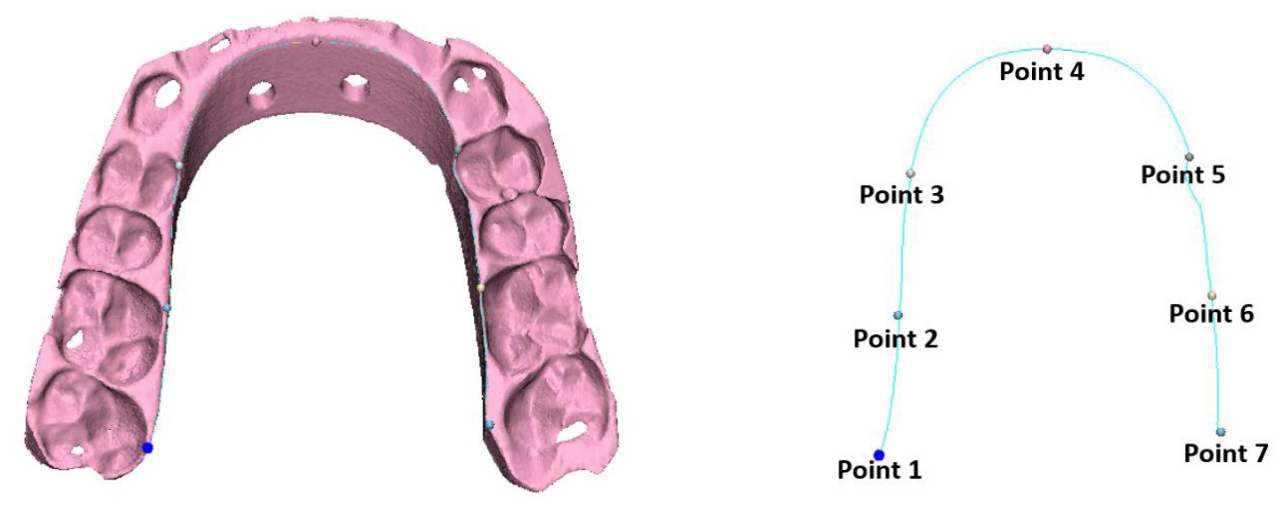
A curve drawn on the pre-sterilized splint and 7 landmarks (points) indicated.

**Table 1:** Results of the volumetric assessment for the TAT, splint and the surgical guide groups.

–	–	Volume (mm^3^)	Percentage Change
TAT	Original	601,7	-
	Pre-sterilization	603,2	+0,2%
	Post-sterilization heat	599,5	-0,6%
	Post-sterilization gas	606,7	+0.6%
Splint	Original	3363,1	-
	Pre-sterilization	3364,5	0,0%
	Post-sterilization heat	3414,8	+1,5%
	Post-sterilization gas	3389,9	+0,8%
Surgical guide	Original	1509,2	-
	Pre-sterilization	1535,7	+1,8%
	Post-sterilization heat	1557,7	+1,4%
	Post-sterilization gas	1548,8	+0,9%

**Table 2:** Results of the morphological assessment for the TAT and the surgical guide groups.

–	–	Mean (mm)	Standard Deviation (mm)	Root Mean Square (mm)
TAT	post-sterilization heat vs pre-sterilization	-0,005	0,036	0,036
	post-sterilization gas vs pre-sterilization	0,009	0,045	0,045
Surgical guide	post-sterilization heat vs pre-sterilization	0,014	0,068	0,069
	post-sterilization gas vs pre-sterilization	0,008	0,086	0,086

**Table 3:** The distances between the 7 landmarks on post-sterilized heat and gas splints to the original curve respectively.

–	Distance to Curve (mm)	
–	Heat Sterilization	Gas Sterilization
Point 1	1,7	0,8
Point 2	1	0,5
Point 3	0,9	0,3
Point 4	0,1	0
Point 5	0,2	0,1
Point 6	0,5	0,3
Point 7	1,5	0,2
